# Supplementary material for: A tool to measure the impact of inaction toward elimination of hepatitis C: A case study in Korea
Source: PLoS One. 2020 Apr 28;15(4):e0232186. doi: 10.1371/journal.pone.0232186 (PMC7188208; doi:10.1371/journal.pone.0232186)
Supplement: Attachment — Submitted filename: Comments PONE D 19 29501.docx [file pone.0232186.s002.docx]

Title; A tool to measure the impact of inaction toward elimination of hepatitis C: A case study in Korea

Authors; Yong Kyun Won, Kyung Sik Kang, Yuri Sanchez Gonzalez, Homie Razavi, Ellen Dugan, Kwang-Hyub Han, Sang Hoon Ahn, Mi Young Jeon, Do Young Kim

Comments

The authors investigated the clinical and economic impact of implementing national screening and treatment policies toward HCV elimination in Korea.

Conclusion of this study is really informative and useful for policy and decision-making.

Although study is well designed and manuscript is clearly organized, there are some points to be improved.

1. Discussion. This study showed that accelerating initiation of interventions to meet elimination targets by 2025 leads to the best profit. Please mention the current issue and how to resolve it.
2. Figures. It is difficult to see the difference because the lines overlap, so please change the scale (aspect ratio) to make it easier to understand.
