# Supplementary material for: A tool to measure the impact of inaction toward elimination of hepatitis C: A case study in Korea
Source: PLoS One. 2020 Apr 28;15(4):e0232186. doi: 10.1371/journal.pone.0232186 (PMC7188208; doi:10.1371/journal.pone.0232186)
Supplement: Attachment — Submitted filename: Response to reviewers_Final.docx [file pone.0232186.s003.docx]

Feb, 2020

Dear Reviewers of Plos One

Thank you for your kind review for enhancing the quality of the manuscript. I and co-authors agree with your points and prepare the revised manuscript and revised figures for re-submission.

From Major comments:

**Comments: The authors set five modelled scenario 1, status quo, 2, standard elimination, 3, delayed elimination by one year, 4, delaying elimination by two years, 5, accelerated elimination.
When using Marcov model, it is important to mention about Quality Adjusted Life Years.
Therefore, they should refer about the prognosis of Hepatocellular carcinoma and end stage of liver cirrhosis.**

🡪 We acknowledged this concern in the Discussions section, line 310. We mention that QALY was not addressed in this study, which is a limitation: “The societal outcomes of these scenarios, such as loss of productivity or quality-adjusted life years, were not considered.”

**Fig1, liver-related death should be divided Hepatocellular carcinoma and end stage of liver cirrhosis independently.**

🡪 We revise Fig1 as advised (Total, HCC, End stage liver Cirrhosis

**Comments: They described a number of HCV-related healthcare-associated outbreaks reported between 2015 and 2016 increased disease recognition and prompted procedural changes.
They should add the number of estimated HCV antibody positive patients in Korea.**

🡪 we add the number of patients in each outbreak in line 237-238.

Discussion. **This study showed that accelerating initiation of interventions to meet elimination targets by 2025 leads to the best profit. Please mention the current issue and how to resolve it**

🡪 For this comment, we added current issues and the way to resolve it in current circumstances of South Korea. Which is mainly located in second half of “discussion”.

**Figures. It is difficult to see the difference because the lines overlap, so please change the scale (aspect ratio) to make it easier to understand.**

🡪 We re-manufactured the graphs more visible and clearer. Please see the new Figure files

**1) Please confirm future researchers can request access to the data underlying your study from the following contact information: (https://ocr.yuhs.ac/HPC/HPCIndex.aspx , Tel:+82-2-2228-0430, E-mail: irb@yuhs.ac). If not, please provide the appropriate contact information where future researchers can send data access queries.**

🡪 (in cover letter) Therefore, the author restricts the right for data access. DY Kim, corresponding author, has obtained approval for this research from his center’s institutional review board (https://ocr.yuhs.ac/HPC/HPCIndex.aspx , Tel:+82-2-2228-0430, E-mail: irb@yuhs.ac). Future researchers can request access to the data underlying your study from DY Kim, Corresponding author (DYK1025@yuhs.ac).

**Additional comment: However, please note PLOS ONE's data availability policy does not require authors to be the sole point of contact for fielding data access queries. Authors must provide a non author point of contact where data requests can be made. Therefore, please provide a non author point of contact where the data requests can be made (such as an institutional email for KNHIS).**

The point of contact where the data requests was done and can be made further in KNHIS is as follow (Mr. HC Yoon, Data operation team #3, Dept. of Big data, Health insurance policy institute, KNHIS., Tel: +82-33-736-2473, (+82-33-811-2000 (English call center for foreigners)) Fax: +82-33-749-6337 E-mail: [sapsary@nhis.or.kr](mailto:sapsary@nhis.or.kr) )

**2) Please also confirm the SAS code has been uploaded as a supporting information file. If it has not been already, please upload it as a supporting information file.**

🡪 (in cover letter) Data from KNHIS was also obtained under this IRB approval. The authors will also attach the SAS code used in the extraction of KNHIS data. The data file analyzed using the code is not provided because it is not open to the public by KNHIS’s regulation.

Hope this revision meeting your pointed issues. Once again, I and co-authors appreciate the kind review and please review our new revised version.

Regards

DY Kim

# Professor. Yonsei University, College of Medicine, Department of Internal Medicine, Seoul, Korea.

# E-mail: DYK1025@yuhs.ac
